# Supplementary material for: Complexome profiling of the Chlamydomonas psb28 mutant reveals TEF5 as an early PSII assembly factor
Source: Plant Cell. 2025 Mar 17;37(6):koaf055. doi: 10.1093/plcell/koaf055 (PMC12164590; doi:10.1093/plcell/koaf055)
Supplement: koaf055_Supplementary_Data [file koaf055_supplementary_data.zip › psb28_interactive.zip!/plots/intensity/qualityPlots/peptides/TIC20_Cre08.g379650.t1.2.html]

### Cre08.g379650.t1.2

Trivial name: TIC20  
SeqCoverage [median]: 6.60  
Mass: 29.52 kDa  
  
 

| Sequence | MissedCleavage | Mass [Da] | wtIntensity | muIntensity | Score |
| --- | --- | --- | --- | --- | --- |
| AEDAEKQDWK | 1 | 1218.55 | 0.00 | 301.47 | 61.56 |
| TVYFVER | 0 | 912.47 | 0.00 | 1300.34 | 68.92 |
|  |  |  | 0.0 | 1601.8 |  |
